# Impact of subdivision of pathological stage I colorectal cancer

**DOI:** 10.1002/ags3.12407

**Published:** 2020-11-11

**Authors:** Shoichi Fujii, Ryu Shimada, Mitsuo Tsukamoto, Tamuro Hayama, Atsushi Ishibe, Jun Watanabe, Takashi Deguchi, Kenji Tsutsumi, Keiji Matsuda, Yojiro Hashiguchi

**Affiliations:** ^1^ Department of Surgery Koga Community Hospital Yaizu Japan; ^2^ Department of Surgery Teikyo University School of Medicine Tokyo Japan; ^3^ Department of Gastroenterological Surgery Yokohama City University Medical Center Yokohama Japan

**Keywords:** colorectal cancer, long‐term outcome, pathological stage I, subdivision of staging, TNM staging

## Abstract

**Aim:**

Stage II‐IV colorectal cancers are subdivided according to TNM categories. However, stage I cases are a single category, despite the inclusion of both T1 and T2 cases, which may have different outcomes. The aim of this study was to evaluate the usefulness of subdividing stage I colorectal cancers by T category.

**Methods:**

From 1984 to 2015, 844 patients with stage I colorectal cancer (T1: 446, T2: 398) underwent colorectal resection with lymph node dissection at three hospitals. The long‐term survival and recurrence rates were compared between T1 and T2. A Cox regression analysis was used to evaluate the risk factors associated with cancer recurrence.

**Results:**

A comparison of the T1 and T2 groups revealed significant differences in 5‐year overall (95.9% vs 91.4%, *P* = .008), recurrence‐free (94.8% vs 87.1%, *P* = .0007), and cancer‐specific survival (97.6% vs 93.6%, *P* = .004), and in the overall (2.5% vs 6.8%, *P* = .003), local (0.2% vs 1.5%, *P* = .04), and lymph node recurrence rates (0.2% vs 1.5%, *P* = .04). All local and lymph node recurrences were associated with lower rectal cancer, and this difference was significant. The Cox multivariate analysis identified male sex (*P* = .01, hazard ratio: 4.00, 95% confidence interval: 1.38‐11.55), T2 (*P* = .02, hazard ratio: 2.98, 95% confidence interval: 1.17‐7.60), and venous invasion (*P* = .03, hazard ratio: 2.38, 95% confidence interval: 1.12‐5.10) as risk factors for recurrence.

**Conclusions:**

The subdivision of stage I colorectal cancer according to T category clearly reflected the long‐term outcomes.

## Graphical abstract

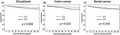

## 1 INTRODUCTION

Colorectal cancers are staged according to the tumor‐node‐metastasis (TNM) system that was developed and updated by the International Union for Cancer Control (UICC). Each revision of this system has included subdivisions of stages II, III, and IV by TNM categories, and all three stages were divided into three classes (A, B, and C) in the 8th edition of the TNM classification.^1^ For colorectal cancers, the subdivisions of stages II and III clearly reflect the prognoses associated with the corresponding T and N categories.^2^ According to the 7th edition of the Cancer Staging Manual, the TNM classification of cancers of the colon and rectum provides more detail than other staging systems. In that edition, stage T4 was subdivided into T4a and T4b, while Stage II was subdivided into three subclasses rather than the binary subdivision used in previous editions.^2^ In the 8th edition, the M category was subdivided into three categories, rather than two, because peritoneal metastasis was subdivided as category M1c, whereas the T and N categories were unchanged.^1^ This latest subdivision clearly demonstrates the different prognoses associated with lesions of each stage.

In contrast, stage I was not subdivided even in this latest edition of the TNM classification. Although the Surveillance, Epidemiology and End Results Program (SEER) reported 5‐year relative survival rates of ≥90% for both T1N0 and T2N0 colorectal cancer cases, a difference of 4.5% in the 5‐year relative survival of rectal cancer was observed between T1 and T2 cases.^2^ The Japanese Society for Cancer of the Colon and Rectum (JSCCR) also reported a significant difference in the recurrence rates between pT1 and pT2 cases (4.0% vs 7.3%, *P* = .0076).^3^ Stage I colorectal cancers in the T1 category can be cured radically even by endoscopic treatment, whereas most cases in the T2 category require bowel resection with lymph node dissection. Moreover, T1 and T2 cases differ significantly in terms of the recurrence rate, according to data from the JSCCR.^3^ These T‐category‐related differences in long‐term outcomes might affect the surveillance schedule after curative resection. These data suggest that accurate prognostic predictions require the subdivision of pStage I cases. With this study, we aimed to clarify the usefulness of subdividing stage I colorectal cancers by examining the long‐term outcomes of patients according to the T category.

## 2 METHODS

### 2.1 Patients

This was a retrospective cohort study of integrated data collected at Koga Community Hospital, Yokohama City University Gastroenterological Center, and Teikyo University. All colorectal cancer patients who underwent curative surgery at these three hospitals between 1984 and 2015 were initially enrolled. The study inclusion criteria were: (i) a histological diagnosis of stage I colorectal cancer according to the TNM system; and (ii) treatment via bowel resection with lymph node dissection. The exclusion criteria were: (i) classification as another stage (0, II, III, or IV); (ii) treatment via irradiation therapy or local excision; (iii) double cancer; (iv) a short follow‐up (<36 months); and (v) incomplete data. The patients were divided into two groups depending on whether they were categorized as pathological (p) T1 or pT2 according to the following definitions: pT1, tumor invasion of the submucosa; pT2, tumor invasion of the muscularis propria. The classification of tumor site was according to the 9th edition of the Japanese Classification of Colorectal Appendiceal and Anal Carcinoma.^4^ The range of rectum was defined from upper edge of puborectal muscle to promontory of sacrum. Upper side of the peritoneal reflection was defined as the upper rectum, and lower side was the lower rectum.

This study protocol was approved by the ethics committees of Koga Community Hospital, Yokohama City University Gastroenterological Center, and Teikyo University. Consent for this study was not received from all patients, because this was a retrospective observational study. However, the information on this study was disclosed on the homepage (http://www.sunkohkai.or.jp/) of our facility. If the patients and their family had an objection to this study, the corresponding data were deleted.

### 2.2 Surveillance

Postoperative surveillance was performed according to the JSCCR guidelines for the treatment of colorectal cancer. The patients were followed through outpatient examinations, including tumor marker measurements every 3 months and chest, abdominal and pelvic computed tomography (CT) scans every 6 months for the first 3 years. These examinations were performed annually from the third to the fifth year. Colonoscopies were performed after 1 and 3 years for patients with colon cancer, and annually for patients with rectal cancer up to the fifth year.

### 2.3 Analyzed parameters

The overall (OS), recurrence‐free (RFS) and cancer‐specific survival (CSS) rates, recurrence rates, and timing were compared between the pT1 and pT2 groups. Furthermore, the survival and recurrence rates were compared in the colon only and the rectal cancer only, respectively, between the pT1 and pT2 groups. The recurrence rates according to the recurrence site were compared between the pT1 and pT2 groups. The causes of death were analyzed between the pT1 and pT2 groups.

The risk factors for recurrence were identified using a multivariate regression analysis. The following items were examined: clinical findings (sex, age, tumor site [colon or rectum], hemoglobin concentration, body mass index, serum carcinoembryonic antigen [CEA] concentration, concomitant disease), perioperative findings (approach [open or laparoscopic], operative time, blood loss volume, postoperative complication, number of dissected lymph nodes) and pathological findings (pathological T category, tumor diameter, histology [tubular adenocarcinoma or mucinous/poorly differentiated adenocarcinoma/signet ring cell carcinoma], lymphatic invasion, venous invasion).

### 2.4 Statistical analysis

The data are presented as medians for continuous variables and as frequencies and percentages (%) for categorical variables. The Mann‐Whitney *U* test and Student's *t*‐test were used to evaluate the significance of differences in continuous variables. The chi‐squared test was used to evaluate the significance of differences in proportions. The survival rates were compared using a Kaplan‐Meier analysis, and significant differences were determined using the log‐rank test. A Cox multivariate regression analysis was used to identify significant risk factors for disease recurrence. In all statistical analyses, a *P*‐value of <0.05 was considered statistically significant. IBM SPSS software for Windows, version 21 (SPSS Inc, Chicago, IL, USA) was used for the statistical analyses.

## 3 RESULTS

### 3.1 Study profile

**FIGURE 1:**
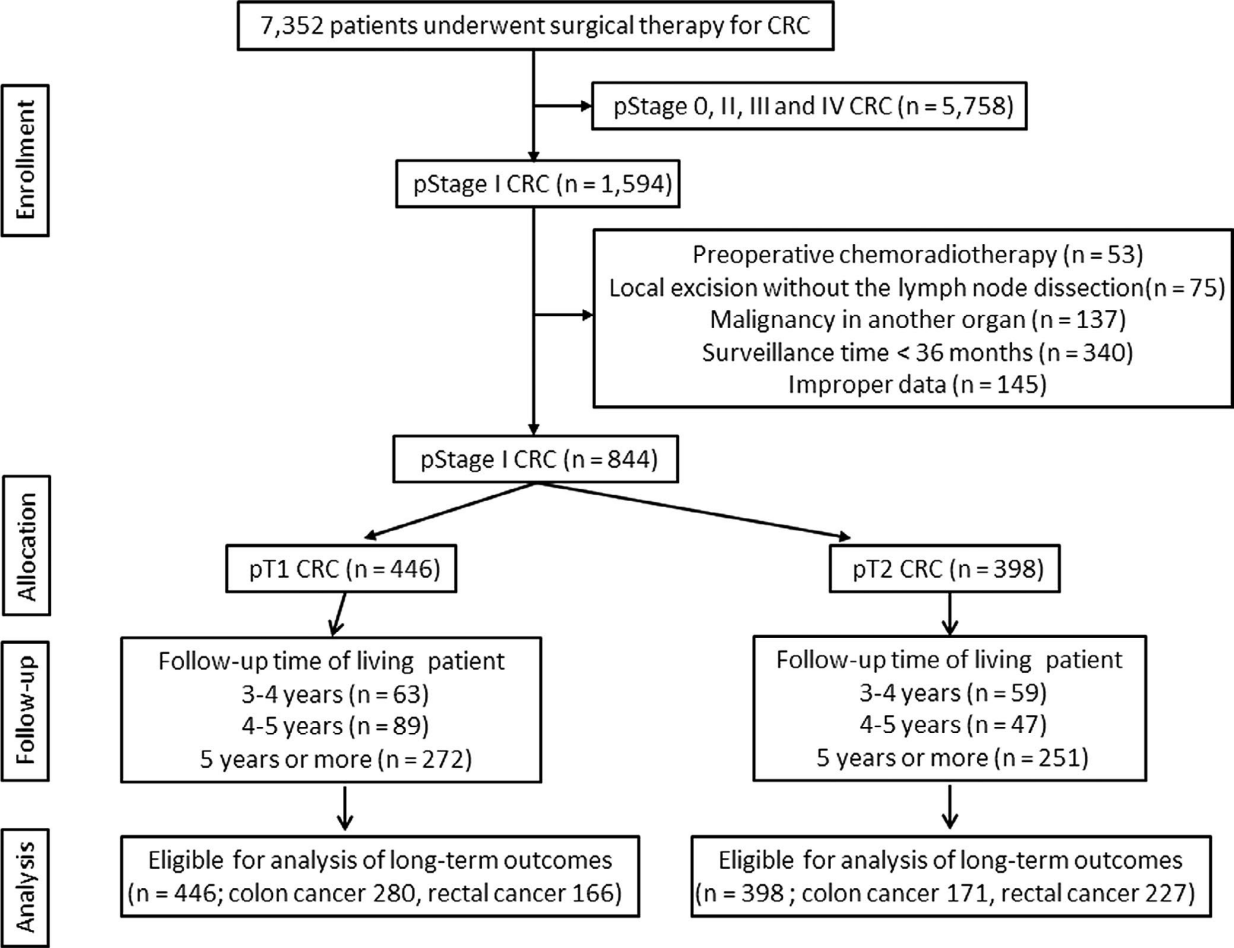
Consort diagram. CRC = Colorectal cancer

Between 1984 and 2015, 7352 patients underwent surgical therapy for primary colorectal cancer at the participating institutions. Of these, 5758 patients with pathological stage 0, II, III, and IV disease were excluded. An additional 749 patients were excluded for the following reasons: chemoradiotherapy, 53 patients; local excision without lymph node dissection, 75 patients; second malignancy in another organ, 137 patients; a follow‐up duration <36 months, 340 patients; and inappropriate data (uncertain pathological stage and prognosis), 145 patients. Finally, 844 patients (pT1: *n* = 446; colon cancer 280, rectal cancer 166, pT2: *n* = 398; colon cancer 171, rectal cancer 227) were included in the study. A CONSORT diagram of this study is shown in Figure 1.

### 3.2 Patients’ characteristics

Some differences were observed in the backgrounds of patients in the pT1 and pT2 groups. Particularly, the pT2 group included a lot of rectal cancers, as well as a higher serum CEA concentration than that in the pT1 group. Regarding the histological findings, the pT2 group had more frequent lymphatic and venous invasion and larger tumor diameters relative to the pT1 group. In the surgical procedure, the rate of laparoscopic surgery in the pT1 group was higher than that in the pT2 group. And, the degree of lymph node dissection in the pT2 group was higher than that in the pT1 group. Regarding the surgical outcomes, patients in the pT2 group had a longer operative time, larger blood loss volume, higher incidence of postoperative complications, and greater number of dissected lymph nodes compared to the pT1 group. There were no inter‐group differences in the surveillance duration or the incidence of surveillance duration ≥5 years (Table 1).

**TABLE 1:** Patients’ characteristics

	pT1 (n = 446)	pT2 (n = 398)	*P‐*value
Age (years, median and range)	64 (31‐89)	65 (29‐90)	.366^a^
Sex (n, %)			
Male	283 (63.5)	232 (58.3)	.125^b^
Female	163 (36.5)	166 (41.7)	
Tumor site (n, %)			
Right colon	75 (16.8)	55 (13.8)	<.001^b^
Transverse colon	38 (8.5)	19 (4.8)	
Left colon	167 (37.4)	97 (24.4)	
Upper rectum	85 (19.1)	122 (30.7)	
Lower rectum	81 (18.2)	105 (26.4)	
Serum CEA concentration (ng/mL, mean ± SD)	2.65 ± 3.61	4.00 ± 6.79	.001^c^
Perioperative findings			
Laparoscopic surgery (n, %)	267 (59.9)	140 (35.2)	<.001^b^
Degree of lymph node dissection (n, %)			
D1	104 (23.3)	35 (8.8)	<.001^b^
D2	180 (40.4)	93 (23.4)	
D3	162 (36.3)	270 (67.8)	
Operative time (minute, mean ± SD)	235 ± 87	264 ± 108	.001^c^
Amount of blood loss (ml, mean ± SD)	200 ± 405	382 ± 679	<.001^c^
Number of lymph node dissection (n, median and range)	18 ± 12	25 ± 16	<.001^c^
Postoperative complication (n, %)	97 (21.7)	114 (28.6)	.021^b^
Pathological findings			
Undifferentiated carcinoma (poorly, mucinous and signet ring cell carcinoma, n, %)	12 (3.0)	22 (5.61)	.062^b^
Lymphatic invasion (n, %)	69 (15.5)	107 (26.9)	<.001^b^
Venous invasion (n, %)	81 (18.2)	117 (29.4)	.001^b^
Tumor diameter (mm, mean ± SD)	22 ± 15	33 ± 14	<.001^c^
Surveillance			
Surveillance time (month, range)	61 (36‐204)	66 (36‐275)	.195^a^
Five years or more surveillance in the living patient (n, %)	272 (64.2)	251 (70.3)	.068^b^

Abbreviation: SD, Standard deviation.

^a^ Mann‐Whitney *U* test.

^b^ Chi‐squared test.

^c^ Student's *t*‐test.

### 3.3 Survival outcomes

**FIGURE 2:**
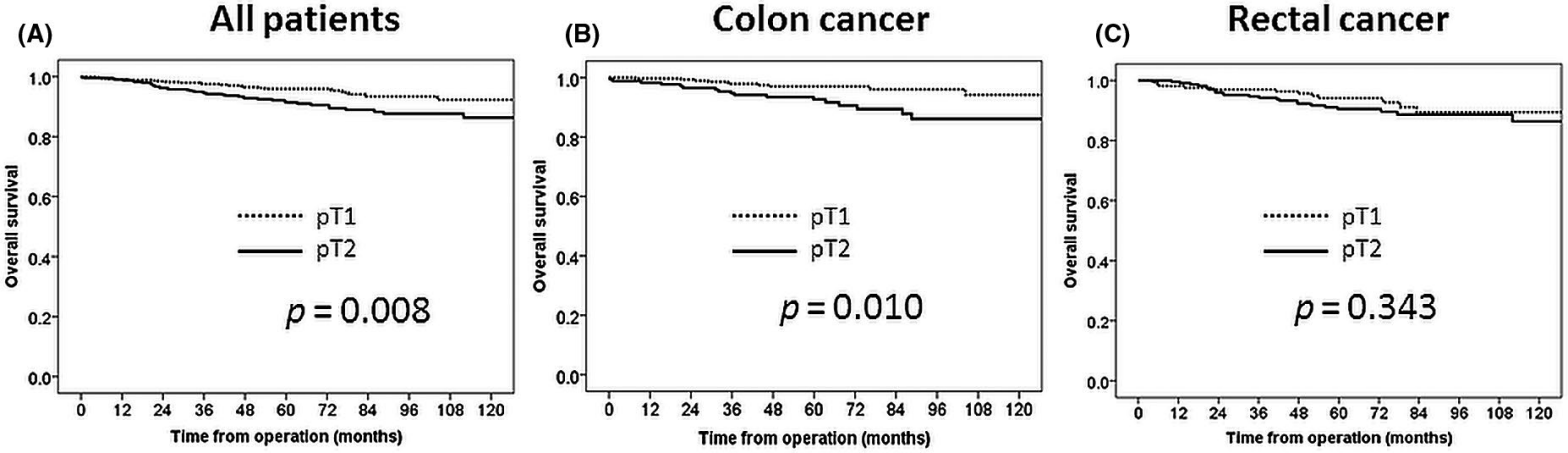
Overall survival. A, All patients; B. Colon cancer; C. Rectal cancer. *P*‐Value was examined by log‐rank test

**FIGURE 3:**
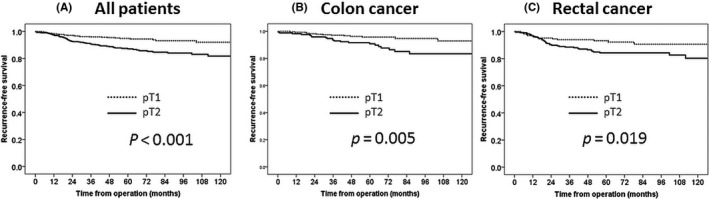
Recurrence‐free survival. A, All patients; B. Colon cancer; C. Rectal cancer. *P*‐Value was examined by log‐rank test

**FIGURE 4:**
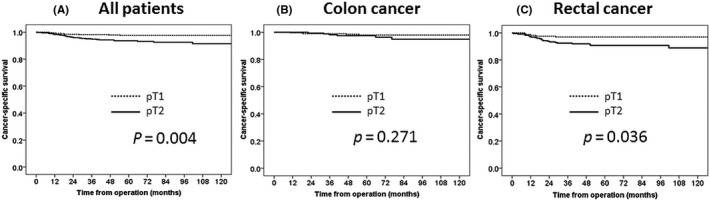
Cancer‐specific survival. A, All patients; B. Colon cancer; C. Rectal cancer. *P*‐Value was examined by log‐rank test

The 5‐year OS rates were 95.9% and 91.4% in the pT1 and pT2 groups, respectively, and this difference was significant (*P* = .008; Figure 2). The 5‐year RFS rates were 94.8% and 87.1% in the pT1 and pT2 groups, respectively, and this difference was significant (*P = *.0007; Figure 3). The 5‐year CSS rates were 97.6% and 93.6% in the pT1 and pT2 groups, respectively, and this difference was significant (*P* = .004; Figure 4).

The survival outcomes of the pT1 and pT2 groups were also compared with respect to the tumor site. There were significant inter‐group differences in the 5‐year OS rate in cases of colon cancer (pT1: 97.0% vs pT2: 92.6%, *P* = .01), the 5‐year RFS rate in cases of colon cancer (95.8% vs 90.8%, *P* = .005) and rectal cancer (93.2% vs 84.3%, *P* = .02), and the 5‐year CSS rate in cases of rectal cancer (96.9% vs 90.7%, *P* = .04).

### 3.4 Recurrences

Among the 844 included patients, 38 (4.5%) were diagnosed with disease recurrences. The recurrence rates were 2.4% (11/451) in colon cancer and 6.9% (27/393) in rectal cancer. The overall recurrence rates were 2.5% and 6.8% in the pT1 and pT2 groups, respectively, and this difference was significant (*P* = .003). However, the groups did not differ in the timing of recurrence (pT1: 35 months vs pT2: 29 months, *P* = .59). Although there was a difference in the recurrence rate for rectal cancer (pT1: 3.6% vs pT2: 9.3%, *P* = .03), no significant difference was detected for colon cancer (1.8% vs 3.5%, *P* = .25).

An analysis according to the recurrence site revealed significant differences in local recurrence (pT1: 0.2% vs pT2: 1.5%, *P* = .04) and lymph node recurrence (0.2% vs 1.5%, *P* = .04; Table 2). All local and lymph node recurrences occurred in patients with lower rectal cancer. All cases of local recurrence occurred in male patients. Four of the seven patients with local recurrence underwent resection of recurrent lesion. Of the seven patients with lymph node recurrence, the inguinal lymph node, pelvic side wall lymph node, and para‐aortic lymph node were affected in three patients, three patients, and one patient, respectively. Three patients with the inguinal lymph node recurrence and one patient with pelvic side wall lymph node recurrence underwent resection of recurrent lesion. The latter patient had been diagnosed with a pT1 lesion and also developed a hepatic recurrence. There were many hepatic metastases in the recurrence of colon cancer (Table 3).

**TABLE 2:** Recurrence sites between pT1 and pT2

n (%)	pT1 (n = 446)	pT2 (n = 398)	*P*‐value
Overall	11 (2.5)	27 (6.8)	.003^b^
Hepatic recurrence	6^a^ (1.3)	9^a^ (2.3)	.315^b^
Pulmonary recurrence	5^a^ (1.1)	8^a^ (2.0)	.295^b^
Peritoneal recurrence	3^a^ (0.7)	0 (0)	.101^b^
Distant lymph node recurrence	1^a^ (0.2)	6^a^ (1.5)	.040^b^
Local recurrence	1 (0.2)	6^a^ (1.5)	.040^b^
Other	1^a^ (0.2)	2^a^ (0.5)	.498^b^

^a^ The number of patients included repetition.

^b^ Chi‐squared test.

**TABLE 3:** Recurrence sites between the colon cancer and the rectal cancer

n (%)	Colon cancer (n = 11)	Rectal cancer (n = 27)	*P*‐value
Hepatic recurrence	8^a^ (72.7)	7^a^ (25.9)	.007^b^
Pulmonary recurrence	4^a^ (36.4)	9^a^ (33.3)	.858^b^
Peritoneal recurrence	2^a^ (18.2)	1 (3.7)	.133^b^
Distant lymph node recurrence	0 (0)	7^a^ (25.9)	.062^b^
Local recurrence	0 (0)	7^a^ (25.9)	.062^b^
Other	2^a^ (8.2)	1^a^ (3.7)	.133^b^

^a^ The number of patients included repetition.

^b^ Chi‐squared test.

### 3.5 Causes of death

Sixty‐three patients (7.5%) died during surveillance, including 22 and 41 patients in the pT1 and pT2 groups, respectively. Six patients (27.3%) in the pT1 group and 10 patients (24.4%) in the pT2 group died of colorectal cancer. Two patients (9.1%) in the pT1 group and five patients (12.2%) in the pT2 group died of other cancer. More than half of these patients in both groups died of non‐cancer diseases (pT1: 14 patients, 63.6% vs pT2: 26 patients, 63.4%). The two groups did not differ significantly in terms of the causes of death (*P* = .918).

According to the analysis of cancer site, four of 27 patients (14.8%) died of the colon cancer and 12 of 36 patients (33.3%) died of the rectal cancer. The number of colorectal cancer deaths was two of 10 patients (20.0%) in the pT1 colon cancer group, two of 17 patients (11.8%) in the pT2 colon cancer group, four of 12 patients (33.3%) in the pT1 rectal cancer group, and eight of 24 patients (33.3%) in the pT2 colon cancer group.

### 3.6 Risk analysis for recurrence

In a univariate regression analysis, the male sex, rectal cancer, serum CEA concentration, pT2 classification, tumor diameter, and venous invasion were identified as risk factors for recurrence. In a multivariate analysis of these six items, male sex (*P* = .01, hazard ratio: 4.00, 95% confidence interval: 1.38‐11.55), pT2 (*P* = .02, hazard ratio: 2.98, 95% confidence interval: 1.17‐7.60), and venous invasion (*P* = .03, hazard ratio: 2.38, 95% confidence interval: 1.12‐5.10) were extracted as significant risk factors for recurrence. The *P*‐value of the rectal cancer was low (*P* = .08), however, it was deleted as a risk factor for recurrence in the multivariate analysis (hazard ratio: 0.47, 95% confidence interval: 0.20‐1.10) (Table 4).

**TABLE 4:** Risk factor for recurrence

Univariate analysis^a^				Multivariate analysis^a^		
*P*‐value		HR	95% CI	*P*‐value	HR	95% CI
Pathological T2	<.01	2.71	1.34‐5.49	.02	2.98	1.17‐7.60
Male sex	<.01	3.61	1.51‐8.62	.01	4.00	1.38‐11.55
Venous invasion	<.01	2.74	1.42‐5.28	.03	2.38	1.12‐5.10
Rectal cancer	<.01	2.83	1.40‐5.71	.08	0.47	0.20‐1.10
Serum CEA concentration	.02	0.04	1.01 ‐1.07	.56	1.01	0.98 ‐1.04
Tumor diameter	.01	1.02	1.01‐1.04	.17	1.02	0.99‐1.04

Abbreviations: CI, confidence interval; HR, Hazard ratio.

^a^ Cox regression analysis.

## 4 DISCUSSION

In our study, we observed differences between the pT1 and T2 groups in every survival analysis. Particularly, the CSS is thought to reflect the malignancy of stage I colorectal cancer, as ≥70% of deaths in both groups were attributed to other diseases. However, we only observed a difference in CSS between pT1 and pT2 cases within the rectal cancer subgroup. We also only observed significant differences in the local and lymph node recurrence rates among patients in the rectal cancer subgroup when we analyzed the recurrent site. Specifically, all local and lymph node recurrences were detected in patients with rectal cancer. Finally, in a multivariate analysis, the T2 category was extracted as a risk factor for recurrence. Our data suggest that it is appropriate to divide stage I into stages Ia and Ib according to the T category.

In the various survival rates for rectal cancer, the OS did not show a significant difference, although the CSS showed a significant difference between T1 and T2. The reason for this discrepancy might be that there were a lot of re‐resections in the local (four of seven patients) recurrence and distant lymph node (four of seven patients) recurrence in the rectal cancer. In contrast, colon cancer did not show a significant difference in the CSS; however, there was a significant difference in the OS between T1 and T2. The reason for the discrepancy of colon cancer survival rates might be that there were a lot of other cancer and disease deaths in pT2 cases in comparison to pT1 cases. The CSS might be more important than the OS because there were a lot of other cancer‐ and disease‐related deaths in the analysis of cause of death. Therefore, the subdivision of T category can be seen to be more meaningful for rectal cancer patients than colon cancer patients. However, rectal cancer was not detected as a risk factor for recurrence in the multivariate analysis. The result of this study cannot conclude that the subdivision of T category is particularly meaningful for rectal cancer. A nationwide re‐inspection by more numbers of patients is necessary.

The subdivision of cancer staging provides an indispensable clinical resource. The subdivision of stages II and III provides important information that informs the selection of postoperative adjuvant chemotherapy. Although the subdivision of stage I might not be useful in this regard because of the high survival rate, it may inform determinations regarding the patient surveillance method. The JSCCR guideline for the treatment of colorectal cancer recommends a tumor marker examination every 3 months and CT scan every 6 months during the first 3 years after surgery for pStage I‐III lesions. After 3 years, the tumor marker examination interval is extended to 6 months, and CT scans are recommended at the same interval until 5 years after surgery.^3^ Our findings suggest that this surveillance schedule might be excessive for pStage I cancers, especially those classified as pT1. A previous Japanese large‐cohort study proposed a stage‐specific surveillance method.^5^ Annual tumor marker evaluations and CT scans might be sufficient for all pT1 colorectal cancers and pT2 colon cancers.

The male sex and venous invasion were extracted as risk factors for the recurrence of pStage I disease through our multivariate regression analysis. We are uncertain why the male sex was identified as a risk factor in our study. Previous studies identified the male sex as a risk factor for anastomotic leakage.^6^, ^7^ Potentially, a narrow pelvis or obesity may lead to technical difficulties when operating on a male patient.

Consistent with our observation, previous studies identified venous invasion as a risk factor for recurrence in T1 colorectal cancer,^8^, ^9^ and vascular invasion as a risk factor for lymph node metastasis in T1 or T2 colorectal cancers.^10^, ^11^, ^12^ Additionally, lymphatic invasion,^13^ poorly differentiated histology,^8^ age, total number of dissected lymph nodes,^14^ preoperative CEA concentration,^12^ and local resection^9^ were also reported as risk factors for recurrence in T1 colorectal cancer. However, those earlier studies also included patients with lymph node metastasis. One previous study analyzed risk factors for mortality in cases of stage I colon cancer identified from the SEER database.^15^ However, only an elevated preoperative serum CEA concentration was predictive of a poor prognosis in that study. Although we similarly identified the serum CEA concentration as a risk factor for recurrence in a univariate regression analysis, this factor was not significant in a multivariate analysis. We attribute this discrepancy between our study and the previous studies’ results to our inclusion of both colon and rectal cancers in the cohort.

Our study had several limitations. First, the investigation period was expanded to approximately 30 years to increase the amount of cumulative data. Consequently, some of the data were old. Advances in treatment during the study period might have led to some natural bias in the data. Second, we observed some differences in the characteristics of patients between the pT1 and pT2 groups that would have influenced the long‐term outcomes, particularly with respect to differences in the tumor site. The inclusion of fewer cases of pT1 rectal cancer than colon cancer in the cohort might indicate that several of the former patients underwent the local excision to avoid the invasiveness of radical curative surgery. Third, our dataset lacks information about some important pathological findings. There were a lot of data losses of tumor budding and perineural invasion. Tumor budding was previously identified as a risk factor for lymph node metastasis.^16^ Specifically, the 2009 JSCCR guidelines for the treatment of colorectal cancer recommend intestinal resection with lymph node dissection as an additional treatment if a budding grade of 2/3 is determined after an endoscopic resection.^3^, ^16^ However, there are no data of budding in our facilities before 2011. There was a loss in more than half of our database. A finding of perineural invasion was identified as a strong prognostic factor in colorectal cancer, similarly.^17^ Our data were old, and neither a staining technique nor pathological diagnostic criteria were uniform. Fourth, the analysis according to the subclassification of pT1 was not done in this study. Depth of submucosal invasive colorectal cancer was reported as an important predictive factor for lymph node metastasis.^18^ Its cut‐off value of distance from muscularis mucosae was 1000 micrometers. In Japan, T1 depth of less than 1000 micrometers was classified to pT1a, and 1000 micrometers or deeper depth of pT1 was classified to pT1b from 2013.^19^ The measuring method of a detailed invasion distance had been described in the Japanese Classification of Colorectal Carcinoma.^4^ However, our pT1 dataset lacks invasion distance about half cases. And, uniform consensus about invasion distance of pT1 tumors has not yet been established internationally. Future studies that include analyses of budding, perineural invasion, international uniformed invasion distance of pT1, and/or other new markers will refine the determination of risk factors associated with pStage I disease.

In conclusion, the subdivision of pStage I according to pT category appeared to provide a good reflection of the long‐term outcomes. We believe that this subdivision into two pStage I classes would be useful for predicting the prognosis of patients and providing effective postoperative surveillance.

## DISCLOSURE

Funding**:** Supported by no foundation.

Conflict of Interest: Shoichi Fujii, Ryu Shimada, Mitsuo Tsukamoto, Tamuro Hayama, Atsushi Ishibe, Jun Watanabe, Takashi Deguchi, Kenji Tsutsumi, Keiji Matsuda, and Yojiro Hashiguchi have no conflict of interest or financial ties to disclose.

Author Contributions: Shoichi Fujii contributed to the study conception and design. Ryu Shimada, Mitsuo Tsukamoto, Tamuro Hayama, Atsushi Ishibe, Jun Watanabe, Takashi Deguchi, Kenji Tsutsumi, and Keiji Matsuda contributed to data acquisition. Shoichi Fujii contributed to the analysis of the data, interpretation of the outcomes, and writing the report. Yojiro Hashiguchi contributed to editing, reviewing, and the final approval of the report.

## References

[1] Amin MB , Edge SB , Greene FL , Byrd DR , Brookland RK , Washington MK , et al. AJCC Cancer Staging Manual, 8th edn. New York, NY: Springer‐Verlag; 2017.

[2] Edge SB , Byrd DR , Compton CC , Fritz AG , Greene F , Trotti A . AJCC Cancer Staging Manual, 7th edn. New York, NY: Springer‐Verlag; 2010.

[3] Hashiguchi Y , Muro K , Saito Y , Ito Y , Ajioka Y , Hamaguchi T , et al. Japanese society for cancer of the colon and rectum (JSCCR) guidelines 2019 for the treatment of colorectal cancer. Int J Clin Oncol. 2020;25:1–42. 31203527 10.1007/s10147-019-01485-z PMC6946738

[4] Japanese Society for Cancer of the Colon and Rectum . Japanese classification of colorectal, appendiceal, and anal carcinoma: 3rd English edition. J Anus Rectum Colon. 2019; 3:175–95. 31768468 10.23922/jarc.2019-018 PMC6845287

[5] Okamura R , Hida K , Nishizaki D , Sugihara K , Sakai Y . Proposal of a stage‐specific surveillance strategy for colorectal cancer patients: a retrospective analysis of Japanese large cohort. Eur J Surg Oncol. 2018;44:449–55. 29397263 10.1016/j.ejso.2018.01.080

[6] Park JS , Choi GS , Kim SH , et al. Multicenter analysis of risk factors for anastomotic leakage after laparoscopic rectal cancer excision: the Korean laparoscopic colorectal surgery study group. Ann Surg. 2013;257:665–71. 23333881 10.1097/SLA.0b013e31827b8ed9

[7] Jannasch O , Klinge T , Otto R , et al. Risk factors, short and long term outcome of anastomotic leaks in rectal cancer. Oncotarget. 2015;6:36884–93. 26392333 10.18632/oncotarget.5170 PMC4742217

[8] Kobayashi H , Mochizuki H , Morita T , Kotake K , Teramoto T , Kameoka S , et al. Characteristics of recurrence after curative resection for T1 colorectal cancer: Japanese multicenter study. J Gastroenterol. 2011;46:203–11. 21152938 10.1007/s00535-010-0341-2

[9] Nam MJ , Han KS , Kim BC , Hong CW , Sohn DK , Chang HJ , et al. Long‐term outcomes of locally or radically resected T1 colorectal cancer. Colorectal Dis. 2016;18:852–60. 26589573 10.1111/codi.13221

[10] Chok KS , Law WL . Prognostic factors affecting survival and recurrence of patients with pT1 and pT2 colorectal cancer. World J Surg. 2007;31:1485–90. 17510767 10.1007/s00268-007-9089-0

[11] Rasheed S , Bowley DM , Aziz O , Tekkis PP , Sadat AE , Guenther T , et al. Can depth of tumour invasion predict lymph node positivity in patients undergoing resection for early rectal cancer? a comparative study between T1 and T2 Cancers. Colorectal Dis. 2008;10:231–8. 18257848 10.1111/j.1463-1318.2007.01411.x

[12] Fang WL , Chang SC , Lin JK , Wang HS , Yang SH , Jiang JK , et al. Metastatic Potential in T1 and T2 Colorectal Cancer. Hepatogastroenterology. 2005;52:1688–91. 16334758

[13] Iida S , Hasegawa H , Okabayashi K , Moritani K , Mukai M , Kitagawa Y . Risk Factors for Postoperative Recurrence in Patients With Pathologically T1 Colorectal Cancer. World J Surg. 2012;36:424–30. 22187130 10.1007/s00268-011-1378-y

[14] Wang HS , Liang WY , Lin TC , Chen W‐S , Jiang J‐K , Yang S‐H , et al. Curative Resection of T1 Colorectal Carcinoma: Risk of Lymph Node Metastasis and Long‐Term Prognosis. Dis Colon Rectum. 2005;48:1182–92. 15793641 10.1007/s10350-004-0935-y

[15] Shen F , Cui J , Hong X , Yu F , Bao X . Preoperative Serum Carcinoembryonic Antigen Elevation in Stage I Colon Cancer: Improved Risk of Mortality in Stage T1 Than Stage T2. Int J Colorectal Dis. 2019;34:1095–104. 31016378 10.1007/s00384-019-03298-y

[16] Ueno H , Mochizuki H , Hashiguchi Y , Shimazaki H , Aida S , Hase K , et al. Risk factors for an adverse outcome in early invasive colorectal carcinoma. Gastroenterology. 2004;127:385–94. 15300569 10.1053/j.gastro.2004.04.022

[17] Knijn N , Mogk SC , Teerenstra S , Simmer F , Nagtegaal ID . Perineural Invasion is a Strong Prognostic Factor in Colorectal Cancer: A Systematic Review. Am J Surg Pathol. 2016;40:103–12. 26426380 10.1097/PAS.0000000000000518

[18] Kitajima K , Fujimori T , Fujii S , Takeda J , Ohkura Y , Kawamata H , et al. Correlations between lymph node metastasis and depth of submucosal invasion in submucosal invasive colorectal carcinoma: a Japanese collaborative study. J Gastroenterol. 2004;39:534–43. 15235870 10.1007/s00535-004-1339-4

[19] Japanese Society for Cancer of the Colon and Rectum . Japanese Classification of Colorectal Carcinoma, 8th edn. Tokyo: Kanehara shuppan; 2013. (in Japanese).

